# ASO Author Reflections: Salvage Surgery for Anal Cancer

**DOI:** 10.1245/s10434-018-7025-1

**Published:** 2018-11-09

**Authors:** J. A. W. Hagemans

**Affiliations:** Department of Surgical Oncology, Erasmus MC Cancer Institute, University Medical Center Rotterdam, Rotterdam, The Netherlands

## Past

Anal squamous cell carcinoma (SSC) is a relatively rare malignancy with an increasing incidence over the last years. Chemoradiotherapy (CRT) has replaced surgery as treatment for primary anal SCC and is currently standard of care for primary anal SCC. Treatment with CRT leads to preservation of the anal sphincter and a 5-year survival rate up to 80%. Failure of CRT occurs in 20–30% of the patients, resulting in persistent or recurrent anal SCC. The only available treatment option to achieve durable local control and survival for persistent or recurrent anal SCC is salvage abdominoperineal resection (APR).^1^ Outcomes of salvage APR for anal SCC were previously described in small and heterogenic groups and with variance in treatment protocols. This study evaluated oncologic outcomes and prognostic factors after salvage APR for anal SCC over almost 3 decades with little change in treatment protocol.

## Present

This study confirmed that salvage APR for either persistent or recurrent anal SCC, after failed initial treatment with CRT, can achieve long-term survival and durable local control.^2^ An overall 5-year survival rate of 41.6% was achieved, and the 5-year local recurrence rate was 44.7%. There was no difference in survival between persistent or recurrent anal SCC. Important prognostic factors associated with decreased survival are an increased pathological tumour size, positive lymph nodes, and involved resection margins. These prognostic factors have been described previously. Achievement of clear resection margins is the most important prognostic factor, which affects survival and local control. This study confirms the benefit of salvage APR for persistent or recurrent anal SCC after failure of primary treatment with CRT. Surgical treatment of re-recurrence after salvage APR, however, does not appear to be useful.

## Future

The use of salvage APR is well established, but achievement of a higher rate of clear resection margins remains a challenge. Intraoperative radiation therapy could be of value to improve overall survival and local control, but further research is warranted.^3^

The biggest challenge remains systemic treatment of unresectable or metastasized anal SCC. Current systemic chemotherapy schemes often are based on 5-FU and Cisplatin and show poor response and survival rates.^4^ However, a recent promising phase-II trial with combined treatment of docetaxel, cisplatin, and fluorouracil for patients with metastatic or unresectable locally recurrent anal SCC showed a high proportion of complete responses and long-term remissions.^5^ Other randomized, controlled trials, including taxanes, targeted therapy, and immunotherapy, are currently performed and could provide promising treatment strategies in the near future. Further investigation should establish the use of these therapies.
